# Perianal Paget's Disease: A Challenge of Surgical Margins in Uncommon Clinical Presentations

**DOI:** 10.7759/cureus.80975

**Published:** 2025-03-21

**Authors:** Sarah K Whitehouse, Heng-Chin Chiam

**Affiliations:** 1 General Surgery, Cairns Hospital, Cairns, AUS

**Keywords:** anal adenocarcinoma, extramammary paget's disease, perianal disease, perianal surgery, primary extramammary paget's disease, surgery in perianal paget's disease

## Abstract

Perianal Paget's disease is a rare entity that often presents with scaly, erythematous plaques. It manifests with an insidious onset, a high frequency of concurrence with malignancy, and difficulties in ensuring clear resection margins. This case highlights the unexpected findings of perianal Paget's disease when completing an excision of invasive adenocarcinoma of the anus. Due to the difficulty with ambiguous borders, wide resection still had involved margins. The patient is currently completing topical treatments.

## Introduction

Paget's disease is an uncommon condition presenting as erythematous plaques on apocrine gland-bearing areas of the body [1]. Extramammary Paget's disease was first described in 1889 and is primarily found in vulval and perineal areas [2,3]. Less than 20% of extramammary Paget's disease occurs in the perianal area [2]. It can be clinically insidious, presenting with nonspecific erythematous or leukoplastic plaques [2,4].

The current standard of treatment for perianal Paget's disease is surgical excision of the lesion [2,4-6]. Many cases require a wide local excision, possibly with the requirement of flap reconstruction or diverting colostomy to promote wound healing [5,7]. Some wide excision cases may be complicated by anal stenosis [7,8]. Difficulty confirming margins is the main reason for a 33%-60% reoccurrence rate in the literature [5]. This can also complicate the re-excision of involved margins if flaps have been used, as it confuses the margins. This has led clinicians to use more radical surgical approaches such as abdominoperineal resection or Mohs micrographic surgery [2]. If surgery is contraindicated or margins are involved, alternative therapies such as imiquimod topical treatments, phototherapy, or radiation therapy have been utilized [2,6,8-12].

## Case presentation

An 86-year-old female patient presented to the General Surgery Outpatients' clinic to investigate a new anal lump, suspected to be a fibroepithelial polyp, associated with bright red per-rectal bleeding. She had no trauma, pain, pruritus, or discharge associated with this lump. She was a frail lady due to advanced age and several health conditions, including atrial fibrillation (on anticoagulation), chronic obstructive pulmonary disease, latent tuberculosis, and hypothyroidism. She had also been investigated for non-specific dermatological issues in other parts of the body, which were severe enough to warrant consideration of immunosuppressive agents.

She underwent a transanal excision of an anal polyp with intraoperative findings documented as a 3 cm polyp at the anal verge at 6 o'clock with a broad stalk. Large hemorrhoids were also noted at 5 and 7 o'clock. Histology unexpectedly showed adenocarcinoma of the anus with involved margins.

After computed tomography (CT), magnetic resonance imaging (MRI), and positron emission tomography (PET) scans showed no concerns for further invasive or metastatic disease, a multidisciplinary team (MDT) meeting recommended a further local resection of the tumor.

**Figure 1:**
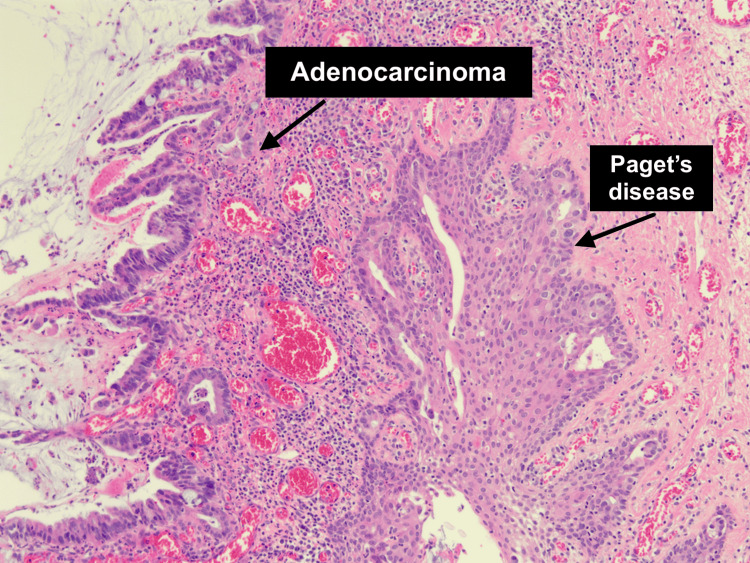
Histology (hematoxylin and eosin stain) slide showing areas of both adenocarcinoma and perianal Paget's disease

**Figure 2:**
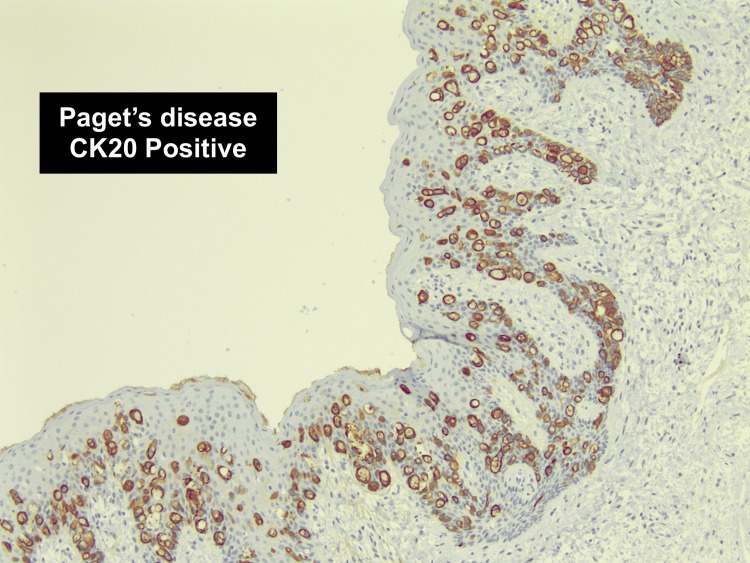
Histology slide (using cytokeratin 20 stain) showing perianal Paget's disease

She underwent a further excision of this lesion, which was completed by a senior surgeon. Intraoperative findings showed no macroscopic lesion or recurrence of the malignant polyp. Hemorrhoids were present at 3, 11, and 7 o'clock, with the latter looking slightly irregular and in continuity with a warty-appearing skin tag. The final excision was from 5 o'clock to 8 o'clock, including both this area and the previous scar. Histology showed a completely excised invasive adenocarcinoma; however, it surprisingly revealed perianal Paget's disease involving the right lateral, left lateral, and distal margins (Figures 1, 2).

**Figure 3:**
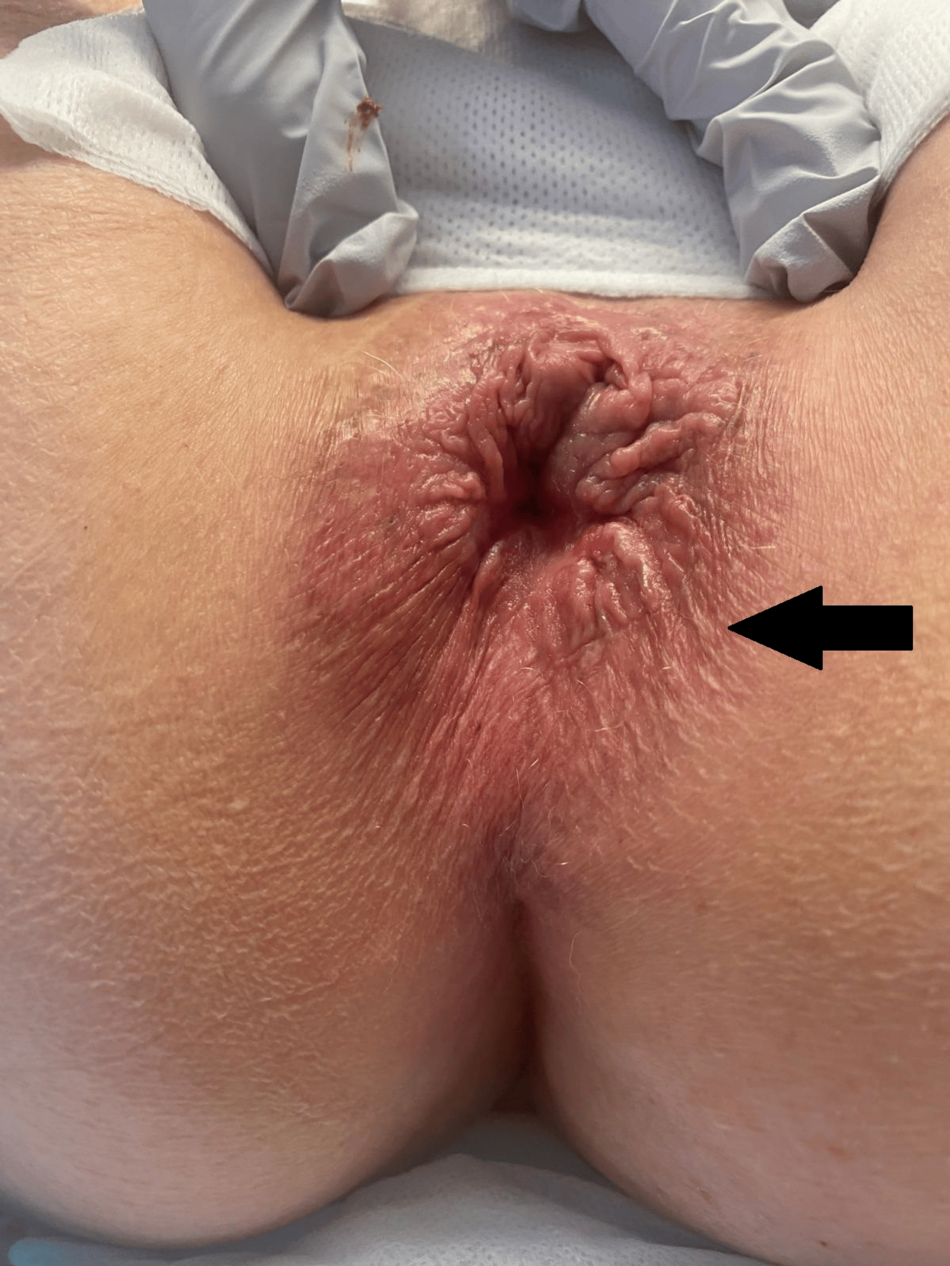
Intraoperative images of the findings of perianal Paget's disease

After re-discussion at MDT, the patient underwent a repeat wide local excision of the previous excision site. Intraoperative findings note a perianal skin lesion at 4 o'clock, 10-15 mm from the anal verge. There were also ill-defined perianal skin changes at 6 o'clock, 10 mm from the anal verge (Figure 3).

An attempt was made to excise this entire area with generous margins. The excision involved 4-9 o'clock areas from 25 mm proximal to the verge to 20 mm radial to the skin. Unfortunately, histology still showed an R1 resection, positive for Paget's disease.

One month postoperatively, she was reviewed in general surgery clinics with an almost healed anal wound and no significant stenosis. Due to the ongoing involvement of margins in a preoperatively frail lady, a decision was made to abandon further attempts at excision and consider topical or photodynamic therapies. Radiation was declined by the patient due to expected side effects.

## Discussion

Perianal Paget’s disease can be a surprise diagnosis, specifically due to its nonspecific and wide-ranging appearance. The link between perianal Paget's and anal or colorectal malignancy is well established but poorly understood due to the rarity of the condition and, thus, difficulty with enough data to analyze [8]. It adds to the body of evidence that would recommend wide-ranging investigation and exclusion of distant malignancies when a diagnosis of Paget's disease is made.

Even large centers, such as the Mayo Clinic or multiple centers, require very long study periods to get small data sets that are unable to guide treatment regimens with great authority [4,5]. Systematic reviews slowly achieve numbers for evidence-based guidelines [9-11].

Due to its association with cancer, targeted cancer screening should be undertaken, depending on its subtype [9,10].

This case, similar to others documented, shows the challenges associated with achieving adequate margin control in this condition. This difficulty and its tendency to occur in older patients leave a question not uncommon to many surgeons: how extensive an operation is too extensive for this patient? Due to our patient's overall frailty, a radical resection with colostomy would likely pose significant risks, both from prolonged anesthetic time and complications related to wound healing and colostomy management. Therefore, with increased risks of anal stenosis, a fourth operation was not undertaken, but management moved to field control.

Further consideration of localized or free flaps could be considered to allow for further wide local excisions. However, the challenge lies in identifying the original border of the excision when positive margins require re-excision. Radiation therapy has been successfully utilized in other cases [12]. Photodynamic therapy and topical immunotherapy have also been used in some cases.

## Conclusions

In summary, this case involves an elderly and comorbid female patient who had a surprise finding of anal adenocarcinoma and Paget's disease of the anus when investigating bright red per-rectal bleeding. She underwent multiple local excisional surgeries to gain local control of the condition. Unfortunately, this was not achieved, and she is undergoing further adjuvant therapy for the area.
